# Identifying the Transportation Needs of a Rural Aging Community: Findings From a Community Assessment

**DOI:** 10.1093/geroni/igaa057.2465

**Published:** 2020-12-16

**Authors:** Vivian Miller, Jordan Wilfong, Melissa Burek, Logan Lanson

**Affiliations:** Bowling Green State University, Bowling Green, Ohio, United States

## Abstract

Community senior centers are tasked with providing aging adults services and programs, congregate meals, and transportation, set forth by the Older Americans Act. The overall function of senior centers is especially critical for rural communities, as rural communities are home to a greater proportion of older adults compared to metropolitan and low-density urban areas. To assess the current needs of the aging population in rural Northwest, OH, a total of 9 focus groups were held (N=45) as part of a larger mixed-methodological study. Through this work, older adults identified limitations of the current senior center transportation. A lack of transportation and accessibility of current transportation were noted as barriers to full senior center use. Findings from this study confirm disparities in rural transportation; a lack of transportation is a major obstacle to program completion. Recommendations address the unique challenges and needs for transportation services for in rural communities are presented.

